# Neurobiological Consequences of Oxidative Stress

**DOI:** 10.2174/1570159X1202140307122223

**Published:** 2014-03

The purpose of this hot topic issue of Current Neuropharmacology is to provide a platform to discuss current knowledge regarding oxidative stress as a contributing/causal factor to the pathophysiology of psychiatric illnesses, including schizophrenia, depression, and anxiety. A better understanding of this mechanism may open new venues for prevention and treatment strategies. Due to the lack of a systematic evaluation of the role of oxidative stress in psychiatric illnesses and given the exciting new findings, this special issue is quite topical. The special issue pulls together investigators who use diverse, but complementary, approaches to study role of oxidative stress in mental health. We have assembled a team of notable experts in the field who utilize complementary approaches to study this aspect. These experts have commented on the current state of knowledge regarding the significance of oxidative stress to mental health and also point towards promising new directions potentially amenable to pharmacological intervention.

*Drs. O’Donnell, Landeira-Fernandez and Salim* have reviewed different behavioral and pharmacological approaches that have been used to examine the outcome of oxidative stress-induced anxiety- and depression-related behaviors. These articles shed light into the novel biochemical pathways critical to the etiology of anxiety and depression that seem highly amenable to therapeutics. *Dr. Michael Maes,* with his expertise in oxidative and nitrosative stress has provided an excellent commentary on how oxidative and nitrosative stress by impacting inflammatory pathways leads to disease. Overall, this hot topic issue provides state-of-the art information on specific genes involved in oxidative stress and the functional significance, and key points for potential intervention. The review article of *Dr. Susan Wood* focusses on central mechanisms that contribute to the psychopathology and cardiovascular disease such as corticotropin releasing factor, neuropeptide Y, monoamines, cytokines and oxidative stress. She has highlighted the neurobiological systems that potentially contribute to the pathophysiology of depression-cardiovascular disease comorbidity. *Dr. Karem Alzoubi* has brought attention to the role of oxidative stress and cognitive impairment while *Dr. Rachel Krolow* has provided an excellent discussion of the role of oxidative stress in anxiety disorders. *Dr. Anil Kumar Pillai*, has added another dimension to this special edition by bringing to the table some of his recent clinical findings on the role of oxidative stress in schizophrenia. 

 Together, we hope these reviews will provide important insights into the molecular pathways underlying the pathogenesis of some of the most debilitating and complex psychiatric ailments. 

